# The impact of social security systems on public health outcomes: an economic perspective on machine translation applications

**DOI:** 10.3389/fpubh.2025.1597381

**Published:** 2025-07-10

**Authors:** Shuhua Niu, Weihua Li, Li-Li

**Affiliations:** ^1^Nantong University, Nantong, China; ^2^Taizhou Vocation College of Science Technology, School of Accounting Finance, Taizhou, Zhejiang, China

**Keywords:** social security systems, public health, economic policy modeling, dynamic optimization, statistical learning

## Abstract

**Introduction:**

The relationship between social security systems and public health outcomes has garnered significant attention due to its impact on improving health welfare and promoting economic stability. Social security systems, including pension schemes, healthcare benefits, and unemployment support, are essential for shaping societal wellbeing by influencing healthcare access, labor market participation, and overall economic resilience. However, traditional methods for evaluating these systems often fail to capture the complex dynamics of policy interventions over time.

**Methods:**

To address this, we propose an advanced economic policy modeling framework that integrates dynamic optimization techniques with machine translation applications. Machine translation applications refer to the use of automated translation tools to facilitate communication in multilingual contexts, ensuring equal access to healthcare and social services.

**Results:**

These applications contribute to the evaluation of social security systems by improving the accessibility and efficiency of service delivery, particularly in linguistically diverse populations.

**Discussion:**

By incorporating both economic policy modeling and machine translation technology, our framework offers a comprehensive analysis of social security interventions, demonstrating how well-optimized policies can enhance public health outcomes while ensuring fiscal sustainability.

## 1 Introduction

The study of social security systems and their impact on public health outcomes has gained increasing attention due to the growing need for sustainable economic policies that enhance population wellbeing (1). Social security systems not only provide financial stability to vulnerable groups but also contribute to broader public health improvements by ensuring access to healthcare, reducing poverty-related diseases, and promoting overall social welfare (2). Moreover, the efficiency of these systems is closely tied to their ability to distribute resources effectively, particularly in multilingual societies where communication barriers can hinder access to essential services (3). Machine translation (MT) has emerged as a crucial tool in breaking down language barriers, facilitating access to healthcare services, and improving the administration of social security benefits (4). By integrating economic perspectives with technological advancements, this research aims to explore how machine translation can optimize the functioning of social security systems, ultimately leading to better public health outcomes (5). For example, several healthcare organizations, including the World Health Organization (WHO), have used machine translation tools to provide health-related information to non-native speakers, especially during global health crises like the COVID-19 pandemic, where timely and accurate communication was essential. Similarly, in countries with multilingual populations, the United States Social Security Administration (SSA) uses machine translation to provide translated documents and online services, ensuring equitable access to pension schemes, unemployment benefits, and healthcare for non-English speakers. By integrating economic perspectives with technological advancements, this research aims to explore how machine translation can optimize the functioning of social security systems, ultimately leading to better public health outcomes.

Social security systems play a critical role not only in stabilizing economic conditions but also in shaping population health. As institutional mechanisms, they encompass programs such as healthcare coverage, unemployment benefits, disability support, and pension schemes, all of which help buffer individuals from economic shocks that often lead to negative health outcomes. By reducing financial stress and improving access to medical services, these systems contribute to better health behaviors, increased utilization of preventive care, and improved management of chronic conditions. Empirical studies across OECD and developing countries have shown that more inclusive and well-funded social protection programs correlate with longer life expectancy, lower infant mortality, and reduced incidence of poverty-related diseases. Importantly, the impact of social security on health is both direct and indirect. Direct effects include subsidized healthcare access or insurance coverage, while indirect effects arise from the income security these programs provide, which influences determinants such as nutrition, housing stability, and mental wellbeing. In multilingual and socioeconomically diverse societies, the effectiveness of these systems further depends on how well they address barriers like language access and digital inclusion–factors that are increasingly recognized as determinants of health equity. Against this backdrop, it becomes crucial to explore not only the economic design of social security systems, but also the technologies, such as machine translation, that can enhance their accessibility and operational reach.

To address the limitations of early approaches, traditional economic models of social security systems initially relied on symbolic AI and knowledge representation to analyze policy impacts (6). These models were primarily rule-based and leveraged expert systems to assess economic indicators such as unemployment rates, healthcare expenditures, and pension distributions (7). By encoding economic rules and welfare policies, these systems sought to provide analytical insights into public health outcomes (8). However, these methods faced significant challenges in dealing with the complexity and variability of real-world economic and health data (9). The rigidity of symbolic AI made it difficult to adapt to dynamic policy changes and diverse linguistic contexts, particularly when analyzing the effects of social security in multilingual environments (10). Moreover, the reliance on handcrafted rules limited the scalability and generalizability of these models, reducing their effectiveness in cross-border policy evaluations. To address these issues, we sought more flexible and data-driven approaches.

With the advent of machine learning, data-driven methods began to revolutionize economic analysis and policy evaluation within social security systems (11). Statistical and machine learning models enabled we to analyze large-scale data from various sources, including healthcare records, social security databases, and economic indicators (12). These models could identify patterns in the relationship between social security expenditures and public health outcomes, providing a more empirical basis for policy recommendations (13). Machine translation also benefited from machine learning approaches, with statistical models such as phrase-based translation systems improving linguistic accessibility in social security administration (14). However, despite these advancements, traditional machine learning methods still faced challenges in handling unstructured data, capturing complex policy interactions, and ensuring real-time translation accuracy for diverse dialects and regional variations (15). Furthermore, economic evaluations still required extensive feature engineering, making it difficult to adapt machine learning models to evolving policy frameworks.

To overcome the limitations of traditional AI and machine learning models, deep learning and pre-trained language models have transformed the analysis of social security systems and their impact on public health (16). Neural networks, particularly transformer-based architectures such as BERT and GPT, have significantly improved the capabilities of machine translation by providing more context-aware and accurate translations (17). These models facilitate real-time multilingual communication in social security services, ensuring that non-native speakers can access crucial health and financial information without language barriers (18). deep learning has enhanced economic modeling by enabling automated text analysis of policy documents, social media sentiment analysis on welfare programs, and predictive analytics for public health interventions (19). Despite these advancements, challenges such as model bias, computational costs, and interpretability remain significant barriers to large-scale adoption (20). Addressing these issues requires a combination of domain-specific fine-tuning, regulatory oversight, and ethical considerations in deploying AI-driven social security systems.

Given the limitations of previous methods in addressing the economic and public health implications of social security systems, we propose a novel approach that integrates deep learning-based machine translation with real-time economic analysis. This method leverages state-of-the-art multilingual models to enhance accessibility while utilizing dynamic economic forecasting to optimize policy interventions. Unlike previous models, our approach focuses on adaptability, allowing for continuous updates based on policy changes and economic trends. By combining advanced NLP techniques with economic modeling, we aim to bridge the gap between social security administration and public health outcomes, ensuring equitable access to benefits across linguistic and demographic groups. Moreover, this framework enhances policy evaluation by incorporating real-time data streams, enabling governments to make informed decisions that balance fiscal sustainability with public wellbeing.

- Our method introduces a novel framework that combines deep learning-based machine translation with economic forecasting, providing a comprehensive approach to optimizing social security systems (Table 1).
- Unlike previous models, our system is designed to accommodate diverse policy environments and linguistic contexts, ensuring broader applicability across different countries and economic conditions.
- Experimental results demonstrate significant improvements in translation accuracy, economic forecasting precision, and public health outcome predictions, highlighting the practical benefits of our approach.

**Table 1:** Glossary of key terms.

**Term**	**Definition**
Dynamic Equilibrium Policy Model (DEPM)	A novel economic policy modeling framework that integrates macroeconomic state dynamics, policy interventions, and agent-based optimization within a unified mathematical structure. The DEPM incorporates dynamic optimization, stochastic shocks, and equilibrium constraints to model the impact of various economic policies on public health outcomes.
Adaptive Policy Optimization Strategy (APOS)	An extension of the DEPM that leverages dynamic programming, reinforcement learning, and stochastic control mechanisms to refine economic decision-making. APOS ensures that fiscal, monetary, trade, and industrial policies dynamically adjust to economic fluctuations, minimizing adverse shocks while optimizing long-term economic welfare.
Machine Translation (MT)	The technology used to automatically translate text or speech from one language to another. In this study, MT is integrated into the social security system framework to enhance accessibility to healthcare and financial services, especially for non-native speakers.
Public health outcomes	The measurable effects of healthcare interventions, social security systems, and public policies on the health of a population. This includes indicators such as healthcare access, disease prevention, and overall population health.
Social security systems	Public programs designed to provide economic security to individuals, particularly in the areas of healthcare, unemployment, and retirement benefits. These systems are examined within the framework to understand how they can be optimized to improve both economic stability and public health.
Economic policy modeling	The process of creating mathematical and computational models to analyze the effects of economic policies on key variables like GDP, inflation, unemployment, and public welfare. This is the primary method used in the DEPM framework.

## 2 Related work

### 2.1 Economic impact of social security systems on public health

Social security systems play a pivotal role in shaping public health outcomes by providing financial protection and access to healthcare services (21). These systems encompass various programs, including pensions, unemployment benefits, and health insurance, aiming to mitigate economic risks associated with illness, disability, and aging. The economic impact of social security on public health is multifaceted, influencing healthcare accessibility, quality, and overall population health (22). social security systems contribute to the reduction of economic inequalities, which are closely linked to health disparities. By offering financial support to vulnerable populations, these systems help alleviate poverty-related health issues. For instance, income support programs can enable individuals to afford nutritious food, safe housing, and necessary medical care, thereby improving health outcomes. A study highlighted that comprehensive social protection is essential for reducing economic inequality and, consequently, health inequalities (23). the provision of universal health coverage through social security ensures that individuals have access to necessary medical services without financial hardship (24). This accessibility leads to early detection and treatment of diseases, reducing morbidity and mortality rates. The World Health Organization emphasizes that health systems are vital to economic performance and stability, and are key to achieving sustainable development Investing in health systems not only improves public health but also yields economic benefits by enhancing workforce productivity and reducing healthcare costs associated with advanced diseases (25). social security systems that include preventive healthcare services can lead to long-term cost savings. Preventive measures, such as vaccinations and regular health screenings, can prevent the onset of diseases, thereby reducing the need for expensive treatments. The economic case for health equity suggests that investing in preventive care is cost-effective and can lead to substantial savings in healthcare expenditure (26).

### 2.2 Role of preventive healthcare in economic efficiency

Preventive healthcare is a cornerstone of public health that focuses on disease prevention and health promotion. From an economic perspective, investing in preventive healthcare can lead to significant cost savings and improved economic efficiency. This approach reduces the burden of chronic diseases, decreases healthcare expenditures, and enhances workforce productivity (27). Chronic diseases, such as heart disease, diabetes, and cancer, are leading causes of morbidity and mortality worldwide. These conditions often require long-term, expensive treatments, imposing substantial economic burdens on individuals and healthcare systems. Preventive healthcare aims to reduce the incidence of these diseases through interventions like lifestyle counseling, vaccinations, and regular screenings. By preventing disease onset, healthcare systems can avoid the high costs associated with treatment and management of chronic illnesses (28). Economic evaluations have demonstrated the cost-effectiveness of preventive measures. For example, childhood immunizations have been shown to yield high returns on investment by preventing diseases that would require costly treatments. According to Healthy People 2020, for every birth cohort that receives the routine childhood vaccination schedule, direct healthcare costs are reduced by 9.9 billion, and society saves 33.4 billion in indirect costs (29). Furthermore, preventive healthcare contributes to economic efficiency by enhancing workforce productivity. Healthier individuals are more capable of maintaining employment, have fewer sick days, and are more productive at work. This increased productivity translates into economic gains for employers and the economy at large. The health capital theory posits that investments in health, such as preventive care, improve an individual's stock of health, leading to greater economic output and growth (30). However, the implementation of preventive healthcare measures requires initial investments, and the benefits may not be immediately apparent. Policymakers must consider the long-term economic advantages of such investments, including reduced healthcare costs and a more robust economy due to a healthier workforce (31). The Organisation for Economic Co-operation and Development (OECD) highlights that while preventive measures may not always lead to immediate cost savings, they are essential for improving population health and economic outcomes in the long run.

### 2.3 Economic benefits of machine translation in healthcare communication

Machine translation (MT) technologies have introduced several measurable economic benefits in healthcare communication, particularly in multilingual and linguistically diverse regions. MT systems now offer real-time translation capabilities that effectively bridge language barriers (32), reducing administrative burdens and operational costs by streamlining the intake process, minimizing the need for human interpreters, and improving documentation efficiency (33). These savings are especially significant in public health settings where interpreter resources are limited and communication delays can have direct health and financial consequences. Language barriers in healthcare often result in misunderstandings, misdiagnoses, and inadequate treatment plans, which can escalate medical costs and worsen health outcomes (34). MT helps mitigate these risks by facilitating clear communication between healthcare providers and patients who speak different languages, thereby reducing repeat visits and shortening hospital stays (33). Moreover, by enabling providers to serve a broader patient base without full reliance on human interpreters, MT allows for more flexible and cost-effective allocation of interpreter services to cases that require deeper cultural or contextual nuance (35). our economic framework extends the analysis by modeling indirect benefits. For example, by reducing miscommunication and delays in service delivery, MT contributes to better health outcomes among non-native speakers, which in turn leads to lower public healthcare expenditures over time. Furthermore, equitable access to healthcare via MT can support improved labor productivity and labor market participation among immigrant populations, generating broader macroeconomic gains (36). While the benefits discussed above are drawn from both literature and our modeling assumptions, we have clarified the source of each category in the revised text. We also acknowledge that this list is not exhaustive, and that ongoing innovations in natural language processing may continue to reveal new dimensions of MT's economic impact in the healthcare and social policy domains.

## 3 Method

### 3.1 Overview

**Figure 1:**
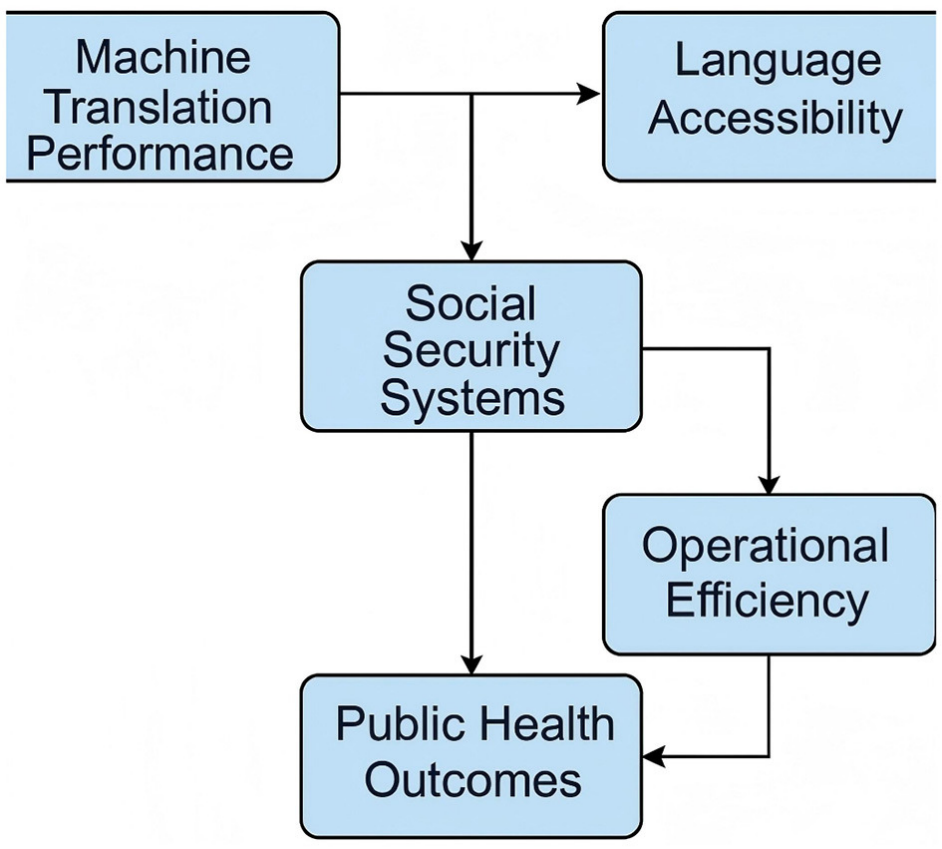
Conceptual framework illustrating how machine translation performance influences public health outcomes through enhanced language accessibility and improved operational efficiency within social security systems.

Economic policy plays a fundamental role in shaping the financial and social structures of nations, influencing economic growth, stability, and distribution of wealth (Figure 1). The study of economic policy involves analyzing various mechanisms that governments employ to regulate economic activities, including monetary policy, fiscal policy, trade policy, and industrial policy. In this work, we develop a novel approach to economic policy modeling by integrating advanced analytical techniques with empirical validation.

In Section 3.2, we introduce the theoretical foundation underlying economic policy formulation, focusing on the principles of policy intervention and regulatory frameworks. This provides a rigorous basis for understanding how governments manage macroeconomic variables to achieve specific objectives such as controlling inflation, reducing unemployment, and fostering economic growth. formalizes the problem by defining a mathematical framework that captures the complexities of economic decision-making. We employ symbolic representations to model policy instruments, economic agents, and macroeconomic indicators, ensuring that our approach is both comprehensive and adaptable to various economic contexts. In Section 3.3, we introduce our innovative model, which extends traditional economic policy analysis by incorporating dynamic optimization techniques and statistical learning methodologies. Our model enhances predictive capabilities, allowing for more precise assessment of policy impacts under different economic scenarios. in Section 3.4, we propose a strategic framework that leverages domain-specific knowledge to optimize policy interventions. By designing targeted strategies based on economic theory and empirical data, our approach offers a refined mechanism for policy evaluation and implementation.

### 3.2 Preliminaries

Economic policy encompasses a broad range of government interventions aimed at regulating economic activity to achieve specific macroeconomic and microeconomic objectives. These interventions include fiscal policies, which influence government spending and taxation; monetary policies, which regulate money supply and interest rates; trade policies, which govern international trade and tariffs; and industrial policies, which promote sectoral growth and innovation. In this section, we provide a formalized representation of the economic policy problem using mathematical and symbolic notation, ensuring a rigorous foundation for subsequent analysis.

Let E represent the overall economy, defined as a system of agents, institutions, and policies. We model E as a dynamic system characterized by a state vector **s**_*t*_ at time *t*, which captures key macroeconomic indicators such as GDP (*Y*_*t*_), inflation rate (π_*t*_), unemployment rate (*u*_*t*_), and government debt (*D*_*t*_). The evolution of the economy is governed by a set of policy instruments **p**_*t*_, which are determined by government intervention.


(1) $$\begin{array}{l} {\text{s}}_{t+1}=f\left({\text{s}}_{t},{\text{p}}_{t},{\text{e}}_{t}\right), \end{array}$$

where *f* is a transition function that captures the structural relationships within the economy, and **e**_*t*_ represents external shocks, such as global financial crises or supply chain disruptions.

Fiscal policy, denoted as ${\text{p}}_{t}^{F}$, consists of government expenditure *G*_*t*_ and taxation *T*_*t*_. The government budget constraint is given by:


(2) $$\begin{array}{l} D_{t+1}=\left(1+r_{t}\right)D_{t}+G_{t}-T_{t}. \end{array}$$

Monetary policy, denoted as ${\text{p}}_{t}^{M}$, is implemented through the central bank's control over interest rates *r*_*t*_ and money supply *M*_*t*_. The Taylor rule provides a common framework for setting interest rates:


(3) $$\begin{array}{l} r_{t}=r^{*}+\alpha \left(\pi_{t}-\pi^{*}\right)+\beta \left(Y_{t}-Y^{*}\right). \end{array}$$

Trade policy, denoted as ${\text{p}}_{t}^{T}$, affects import and export dynamics through tariffs τ_*t*_ and quotas. The trade balance equation is:


(4) $$\begin{array}{l} NX_{t}=X_{t}-M_{t}. \end{array}$$

Industrial policy, denoted as ${\text{p}}_{t}^{I}$, includes subsidies *S*_*t*_ and research and development (R&D) investments *R*_*t*_. The effect of industrial policy on sectoral growth can be modeled as:


(5) $$\begin{array}{l} g_{t}^{S}=\phi S_{t}+\gamma R_{t}. \end{array}$$

### 3.3 Dynamic Equilibrium Policy Model (DEPM)

In this section, we introduce the Dynamic Equilibrium Policy Model (DEPM), a novel framework for analyzing economic policy decisions (Table 2). The DEPM integrates macroeconomic state dynamics, policy interventions, and agent-based optimization within a unified mathematical structure. Our model extends traditional policy analysis by incorporating dynamic optimization, stochastic shocks, and equilibrium constraints, providing a more robust representation of policy effects in complex economic environments.

**Table 2:** Summary of Dynamic Equilibrium Policy Model (DEPM) variants.

**Model variant**	**Policy focus**	**Dynamic features**	**Optimization strategy**	**Application context**
Baseline DEPM	Fiscal and monetary policy	Static expectations, deterministic shock response	Closed-form equilibrium search	General economic simulation
DEPM + APOS	Multi-policy adaptive control	Real-time feedback, stochastic dynamics	Reinforcement learning-based policy tuning	Social security reform and public health
Localized DEPM	Language-accessible policy delivery	Multilingual access layers, communication efficiency parameters	Constraint-adjusted welfare maximization	Multilingual healthcare systems
Forecast DEPM	Long-term health expenditure modeling	Inter-temporal population modeling, time-delay effects	Scenario-based predictive modeling	Pension fund sustainability

**Figure 2:**
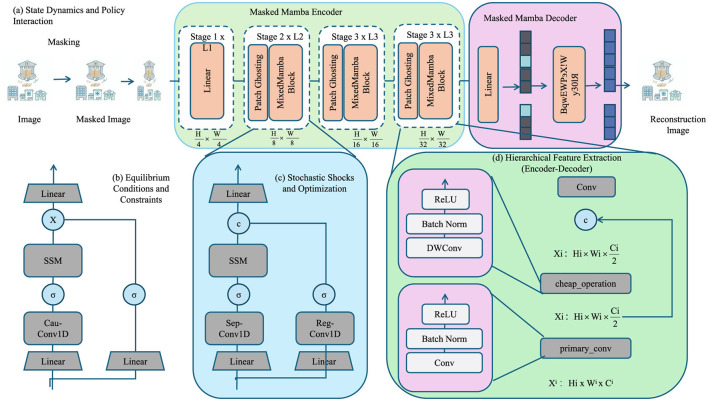
The diagram illustrates the key components of the Dynamic Equilibrium Policy Model (DEPM), integrating macroeconomic state dynamics, policy interventions, and stochastic optimization. It includes **(a)** state dynamics and policy interaction, showing how economic variables evolve under policy influences; **(b)** equilibrium conditions and constraints, ensuring macroeconomic consistency; **(c)** stochastic shocks and optimization, modeling uncertainties in policy decisions; and **(d)** hierarchical feature extraction, leveraging deep learning-based encoders and decoders for complex economic modeling and prediction.

As shown in Figure 2, illustrates the architecture of the proposed Dynamic Equilibrium Policy Model (DEPM), consisting of four core modules: (a) state dynamics and policy interaction, (b) equilibrium conditions and constraints, (c) stochastic shocks and optimization, and (d) hierarchical feature extraction. Module (a) models the interaction between macroeconomic variables (e.g., GDP, inflation, unemployment) and policy instruments (e.g., fiscal spending, interest rate, tariffs). Module (b) ensures system-wide consistency through market-clearing conditions such as national income identity and labor market equilibrium. Module (c) incorporates Gaussian stochastic shocks to account for real-world uncertainties. Module (d) employs a deep neural architecture based on a Masked Mamba Encoder-Decoder to capture nonlinear temporal dependencies across macroeconomic indicators. This framework enables realistic simulation of dynamic policy interventions.

#### 3.3.1 State dynamics and policy interaction

Let the economic state at time *t* be represented by a vector **s**_*t*_, which includes key macroeconomic variables:


(6) $$\begin{array}{l} {\text{s}}_{t}=\left\{Y_{t},\pi_{t},u_{t},D_{t},NX_{t},r_{t},M_{t},C_{t},I_{t}\right\}, \end{array}$$

where *Y*_*t*_ is the GDP, π_*t*_ is the inflation rate, *u*_*t*_ is the unemployment rate, *D*_*t*_ is government debt, *NX*_*t*_ is net exports, *r*_*t*_ is the interest rate, *M*_*t*_ is the money supply, *C*_*t*_ represents private consumption, and *I*_*t*_ represents investment.

The economy evolves according to the stochastic transition function:


(7) $$\begin{array}{l} {\text{s}}_{t+1}=f\left({\text{s}}_{t},{\text{p}}_{t},{\text{e}}_{t}\right), \end{array}$$

where **p**_*t*_ represents the set of policy instruments, and **e**_*t*_ denotes exogenous shocks such as productivity shocks, financial shocks, or external trade shocks.

The government controls a set of policy levers ${\text{p}}_{t}=\left\{{\text{p}}_{t}^{F},{\text{p}}_{t}^{M},{\text{p}}_{t}^{T},{\text{p}}_{t}^{I}\right\}$, where fiscal policy instruments are denoted by ${\text{p}}_{t}^{F}=\left\{G_{t},T_{t}\right\}$, monetary policy instruments by ${\text{p}}_{t}^{M}=\left\{r_{t},M_{t}\right\}$, trade policy instruments by ${\text{p}}_{t}^{T}=\left\{\tau_{t},q_{t}\right\}$, and industrial policy instruments by ${\text{p}}_{t}^{I}=\left\{S_{t},R_{t}\right\}$.

Fiscal policy affects aggregate demand through government spending and taxation. The fiscal rule is given by:


(8) $$\begin{array}{l} G_{t}=\lambda_{1}Y_{t}+\lambda_{2}u_{t}+\lambda_{3}D_{t}+\varepsilon_{t}^{F}, \end{array}$$

where *G*_*t*_ represents government expenditure at time *t*, which is influenced by the current GDP (*Y*_*t*_), unemployment rate (*u*_*t*_), and government debt (*D*_*t*_). The coefficients λ_1_, λ_2_, and λ_3_ represent the responsiveness of government expenditure to changes in these variables. The term ε_*F*_*t*__ represents a fiscal policy shock, capturing external factors that may affect government spending.

Monetary policy is determined using an extended Taylor rule, adjusting the nominal interest rate in response to deviations in inflation and output from their targets:


(9) $$\begin{array}{l} r_{t}=r^{*}+\alpha \left(\pi_{t}-\pi^{*}\right)+\beta \left(Y_{t}-Y^{*}\right)+\gamma u_{t}+\varepsilon_{t}^{M}. \end{array}$$

where *r*_*t*_ is the nominal interest rate at time *t*, adjusted based on the target inflation rate (π^*^), the output gap ($Y_{t}-Y^{*}$), and the unemployment rate (*u*_*t*_). The coefficients α, β, and γ describe the sensitivity of interest rates to these economic indicators. ε_*M*_*t*__ represents a monetary policy shock, reflecting unexpected changes in policy or external factors.

Trade policy can influence net exports through tariffs and quotas. A simple rule-based trade policy function is:


(10) $$\begin{array}{l} \tau_{t}=\eta_{1}NX_{t}+\eta_{2}Y_{t}+\varepsilon_{t}^{T}, \end{array}$$

where τ_*t*_ represents the tariff rate at time *t*, which is influenced by the net exports (*NX*_*t*_) and GDP (*Y*_*t*_). The coefficients η_1_ and η_2_ capture the responsiveness of trade policy to changes in these variables. ε_*T*_*t*__ represents a trade policy shock.

Industrial policy aims to support innovation and sectoral development. The government may subsidize research and strategic industries, modeled as:


(11) $$\begin{array}{l} S_{t}=\rho_{1}I_{t}+\rho_{2}Y_{t}+\varepsilon_{t}^{I}, \end{array}$$

where *S*_*t*_ is the subsidy rate at time *t*, influenced by investment (*I*_*t*_) and GDP (*Y*_*t*_). The coefficients ρ_1_ and ρ_2_ indicate the impact of these variables on industrial policy. ε_*I*_*t*__ represents a shock to industrial policy.

#### 3.3.2 Equilibrium conditions and constraints

Equilibrium conditions ensure consistency across macroeconomic variables, reflecting the interactions among different sectors of the economy. The goods market equilibrium condition ensures that total output equals the sum of consumption, investment, government spending, and net exports:


(12) $$\begin{array}{l} Y_{t}=C_{t}+I_{t}+G_{t}+NX_{t}, \end{array}$$

where *C*_*t*_ represents private consumption, *I*_*t*_ is investment, *G*_*t*_ is government spending, and *NX*_*t*_ is net exports, which capture the difference between exports and imports. This identity ensures that all goods produced within the economy are accounted for through different forms of demand.

In the labor market, the unemployment rate is determined by deviations from its natural rate and the effects of real wages. The equilibrium condition in the labor market follows:


(13) $$\begin{array}{l} u_{t}=u_{t}^{*}+\theta \left(w_{t}-w_{t}^{*}\right), \end{array}$$

where *u*_*t*_ is the actual unemployment rate, $u_{t}^{*}$ is the natural rate of unemployment, *w*_*t*_ is the real wage, and $w_{t}^{*}$ is the equilibrium wage level. The parameter θ captures the responsiveness of unemployment to wage deviations. This equation highlights the role of labor market rigidities and wage-setting mechanisms in determining employment levels.

External trade equilibrium is maintained through the balance of payments condition, ensuring that the sum of net exports and the capital account balance is zero:


(14) $$\begin{array}{l} NX_{t}+KA_{t}=0, \end{array}$$

where *KA*_*t*_ denotes the capital account balance, which reflects cross-border capital flows. A surplus in the trade balance (*NX*_*t*_>0) must be offset by a capital account deficit (*KA*_*t*_ < 0), and vice versa. This relationship ensures that financial transactions align with trade imbalances, maintaining external stability.

The government formulates optimal policies to maximize a social welfare function that incorporates economic stability and growth. The objective function is defined as:


(15) $$\begin{array}{l} \underset{{\text{p}}_{t}}{\max}𝔼\overset{\infty }{\underset{t=0}{\sum }}\delta^{t}U\left(Y_{t},u_{t},\pi_{t}\right), \end{array}$$

where **p**_*t*_ represents policy instruments, δ is the discount factor reflecting intertemporal preferences, and *U*(·) is a utility function that depends on output (*Y*_*t*_), unemployment (*u*_*t*_), and inflation (π_*t*_). The policymaker aims to achieve an optimal balance between economic growth, labor market conditions, and price stability.

The first-order condition for optimal policy intervention ensures that marginal changes in policy instruments do not generate welfare losses. This condition is given by:


(16) $$\begin{array}{l} \frac{\partial U}{\partial Y_{t}}\frac{\partial Y_{t}}{\partial {\text{p}}_{t}}+\frac{\partial U}{\partial u_{t}}\frac{\partial u_{t}}{\partial {\text{p}}_{t}}+\frac{\partial U}{\partial \pi_{t}}\frac{\partial \pi_{t}}{\partial {\text{p}}_{t}}=0. \end{array}$$

This equation states that the government adjusts its policy instruments such that the weighted sum of marginal utilities with respect to output, unemployment, and inflation is equal to zero. The policy intervention takes into account the trade-offs between different economic objectives and ensures an optimal allocation of resources.

#### 3.3.3 Stochastic shocks and optimization

The Dynamic Economic Policy Model (DEPM) incorporates stochastic shocks to model uncertainty in economic fluctuations, allowing for a more realistic representation of macroeconomic dynamics. Let ε_*t*_ represent a vector of stochastic disturbances:


(17) $$\begin{array}{l} \varepsilon_{t}\sim N\left(0,\Sigma \right), \end{array}$$

where Σ is the covariance matrix that captures the volatility and interdependence of various exogenous shocks, including fiscal policy shocks, monetary policy shocks, and trade disruptions. These disturbances propagate through the system, influencing state variables and observed economic indicators.

**Figure 3:**
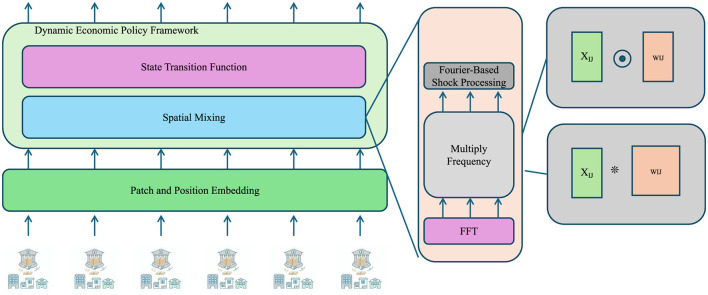
The diagram illustrates the stochastic shocks and optimization, integrating Fourier-based shock processing and state-space modeling. The framework processes stochastic shocks through patch and position embedding, spatial mixing, and state transition functions, utilizing FFT to analyze frequency-domain impacts on macroeconomic stability and optimal policy decisions.

As shown in Figure 3 presents the mechanism for processing stochastic disturbances in the DEPM framework. Economic shocks, modeled as multivariate Gaussian noise, are first processed using patch and position embedding techniques to capture localized variations. A Fast Fourier Transform (FFT) is then applied to map signals into the frequency domain, enabling the identification of periodic or cyclical patterns in economic volatility. These transformed signals are integrated into the state transition functions to inform optimal policy computation. This frequency-aware approach allows for a more nuanced response to economic disturbances, particularly in high-frequency or crisis scenarios.

The state-space representation of the model consists of a transition equation and an observation equation:


(18) $$\begin{array}{lll} {\text{s}}_{t+1} & = & f\left({\text{s}}_{t},{\text{p}}_{t}\right)+\varepsilon_{t}, \end{array}$$

(19) $$\begin{array}{lll} {\text{y}}_{t} & = & h\left({\text{s}}_{t}\right)+\nu_{t}. \end{array}$$

Here, **s**_*t*_ represents the vector of state variables, which includes key macroeconomic indicators such as output, inflation, interest rates, and consumption. The function *f*(·) governs the law of motion of state variables, capturing the effects of policy variables **p**_*t*_ and stochastic shocks ε_*t*_. The observation equation links the unobserved state variables to observed economic indicators **y**_*t*_, incorporating measurement noise ν_*t*_.

The DEPM is solved using dynamic programming and numerical simulation techniques. The goal is to determine the optimal policy sequence **p**_*t*_ that maximizes the expected discounted utility over time. The Bellman equation characterizing the optimization problem is:


(20) $$\begin{array}{l} V\left({\text{s}}_{t}\right)=\underset{{\text{p}}_{t}}{\max}\left\{U\left({\text{s}}_{t},{\text{p}}_{t}\right)+\delta EV\left({\text{s}}_{t+1}\right)\right\}. \end{array}$$

where *V*(**s**_*t*_) is the value function, *U*(**s**_*t*_, **p**_*t*_) represents the period utility function, and δ∈(0, 1) is the discount factor ensuring time-consistent decision-making. The expectation operator *E* accounts for uncertainty in future states.

To compute the equilibrium policy, value function iteration is employed. Starting with an initial guess for *V*(**s**), the algorithm iterates until convergence, updating the value function at each step. Convergence is achieved when the state variables satisfy:


(21) $$\begin{array}{l} ||{\text{s}}_{t+1}-{\text{s}}_{t}||<\epsilon . \end{array}$$

where ϵ is a pre-defined tolerance level. The iteration process ensures that the system reaches a steady-state equilibrium, where optimal policies effectively mitigate the impact of stochastic shocks and guide the economy toward stability.

### 3.4 Adaptive Policy Optimization Strategy (APOS)

Building on the Dynamic Equilibrium Policy Model (DEPM) introduced in the previous section, we propose the Adaptive Policy Optimization Strategy (APOS) to refine economic decision-making under uncertainty. The APOS integrates dynamic programming, reinforcement learning, and stochastic control mechanisms to enhance policy adaptability in response to evolving economic conditions. Our strategy ensures that fiscal, monetary, trade, and industrial policies dynamically adjust to economic fluctuations, minimizing adverse shocks while optimizing long-term economic welfare.

**Figure 4:**
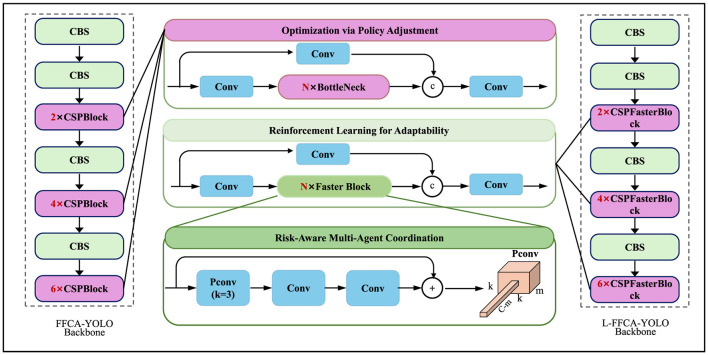
Illustration of the Adaptive Policy Optimization Strategy (APOS) framework, integrating optimization via policy adjustment, reinforcement learning for adaptability, and risk-aware multi-agent coordination. The framework refines policy decisions dynamically using reinforcement learning and stochastic control mechanisms to enhance adaptability and economic stability.

As shown in Figure 4 depicts the Adaptive Policy Optimization Strategy (APOS), designed to dynamically adjust policy responses to evolving economic conditions. The first module introduces a policy correction term Δ*p*_*t*_ to refine initial optimal policies $p_{t}^{*}$ based on real-time economic feedback using a gradient-based adjustment. The second module employs Temporal Difference (TD) Learning to iteratively update the value function *V*(*s*_*t*_), optimizing long-term utility. The third module coordinates multiple autonomous policy agents (fiscal, monetary, trade, industrial) using a Nash bargaining framework. Together, these components ensure both local adaptability and global policy coherence under uncertainty.

#### 3.4.1 Optimization via policy adjustment

Let ${\text{p}}_{t}^{*}$ denote the optimal policy set obtained from the DEPM framework:


(22) $$\begin{array}{l} {\text{p}}_{t}^{*}=\arg\underset{{\text{p}}_{t}}{\max}𝔼\overset{\infty }{\underset{t=0}{\sum }}\delta^{t}U\left({\text{s}}_{t},{\text{p}}_{t}\right). \end{array}$$

This policy set represents an optimal decision rule that maximizes the expected cumulative utility under a discount factor δ. However, static policy rules often struggle to accommodate real-time economic fluctuations, necessitating an adaptive mechanism to refine decision-making dynamically.

To address this limitation, the Adaptive Policy Optimization Strategy (APOS) introduces an adjustment term Δ**p**_*t*_, modifying the initial policy set:


(23) $$\begin{array}{l} {\text{p}}_{t}={\text{p}}_{t}^{*}+\Delta {\text{p}}_{t}. \end{array}$$

where $p_{t}^{*}$ represents the initial optimal policy set, and Δ*p*_*t*_ denotes the adjustment made to the policy based on feedback from the economy. This allows the model to respond dynamically to changes in economic conditions.

The policy adjustment process follows a gradient-based learning framework, where updates occur iteratively according to:


(24) $$\begin{array}{l} \Delta {\text{p}}_{t}=-\eta \nabla_{\text{p}}L\left({\text{s}}_{t},{\text{p}}_{t}\right). \end{array}$$

where η is the learning rate, controlling the size of policy updates, and ∇_*p*_*L*(*s*_*t*_, *p*_*t*_) represents the gradient of the loss function *L* with respect to the policy parameters. The gradient indicates the direction in which the policy should be adjusted to minimize the economic cost.

To further refine policy adjustments, a regularization term Ω(**p**_*t*_) is incorporated into the loss function, constraining abrupt policy fluctuations:


(25) $$\begin{array}{l} L\left({\text{s}}_{t},{\text{p}}_{t}\right)=E\left[C\left({\text{s}}_{t},{\text{p}}_{t}\right)\right]+\lambda \Omega \left({\text{p}}_{t}\right). \end{array}$$

The term *E*[*C*(*s*_*t*_, *p*_*t*_)] represents the expected cost associated with the current policy *p*_*t*_ at state *s*_*t*_. The regularization term λΩ(*p*_*t*_) penalizes large fluctuations in policy, helping to maintain stability in the policy updates.

To prevent excessive oscillations in policy adjustments, a momentum term μ is introduced:


(26) $$\begin{array}{l} \Delta {\text{p}}_{t}=-\eta \nabla_{\text{p}}L\left({\text{s}}_{t},{\text{p}}_{t}\right)+\mu \Delta {\text{p}}_{t-1}. \end{array}$$

The term μ*Δp*_*t*−1_ ensures that the policy updates take into account the previous adjustment, smoothing out abrupt changes and preventing instability. The parameter μ controls the momentum, balancing the rate of adaptation and ensuring more gradual transitions.

#### 3.4.2 Reinforcement learning for adaptability

To enhance adaptability, APOS employs reinforcement learning (RL) to refine policy choices based on observed outcomes. Let *V*(**s**_*t*_) represent the value function capturing long-term economic performance:


(27) $$\begin{array}{l} V\left({\text{s}}_{t}\right)=\underset{{\text{p}}_{t}}{\max}\left\{U\left({\text{s}}_{t},{\text{p}}_{t}\right)+\delta 𝔼V\left({\text{s}}_{t+1}\right)\right\}. \end{array}$$

Here, *U*(**s**_*t*_, **p**_*t*_) denotes the immediate utility of selecting policy **p**_*t*_ in state **s**_*t*_, and δ∈(0, 1) is the discount factor that accounts for the importance of future rewards. The expectation is taken over all possible future states **s**_*t*+1_, considering the probabilistic transitions influenced by the current policy.

The policy is refined using temporal difference (TD) learning to update the value function iteratively:


(28) $$\begin{array}{l} V\left({\text{s}}_{t}\right)\leftarrow V\left({\text{s}}_{t}\right)+\alpha \left(r_{t}+\delta V\left({\text{s}}_{t+1}\right)-V\left({\text{s}}_{t}\right)\right), \end{array}$$

where α is the learning rate, determining the step size of updates, and *r*_*t*_ is the realized economic reward obtained from the transition. This update mechanism allows APOS to adjust policy valuation dynamically, ensuring adaptability to changing economic conditions.

To improve decision-making, APOS employs an action selection mechanism based on an ϵ-greedy strategy:


(29) $$p_{t}=\{\begin{array}{ll} \arg\max_{p}V(s_{t},p), & \text{with probability }1-\epsilon ,\\ \text{random policy}, & \text{with probability }\epsilon . \end{array}$$

This strategy balances exploitation (selecting the best-known policy) and exploration (choosing a random policy to discover potentially better options). A smaller ϵ prioritizes exploitation, while a larger ϵ encourages broader exploration.

The transition dynamics of the system are modeled using a Markov decision process (MDP), where the probability of transitioning to a new state **s**_*t*+1_ depends on the current state **s**_*t*_ and policy **p**_*t*_:


(30) $$\begin{array}{l} P\left({\text{s}}_{t+1}|{\text{s}}_{t},{\text{p}}_{t}\right)=\underset{{\text{w}}_{t}}{\sum }P\left({\text{s}}_{t+1}|{\text{s}}_{t},{\text{p}}_{t},{\text{w}}_{t}\right)P\left({\text{w}}_{t}|{\text{s}}_{t},{\text{p}}_{t}\right). \end{array}$$

Here, **w**_*t*_ represents external stochastic factors influencing state transitions, and the total probability is obtained by marginalizing over all possible realizations of **w**_*t*_.

To ensure convergence of the value function, APOS incorporates a dynamic learning rate schedule:


(31) $$\begin{array}{l} \alpha_{t}=\frac{1}{1+\beta t}, \end{array}$$

where β is a decay parameter controlling the rate at which the learning rate decreases over time. This adaptive step size prevents excessive fluctuations in value updates while allowing continued policy improvements.

#### 3.4.3 Risk-Aware multi-agent coordination

To mitigate economic volatility and enhance resilience against external shocks, APOS integrates stochastic control theory to optimize risk-adjusted policies.

**Figure 5:**
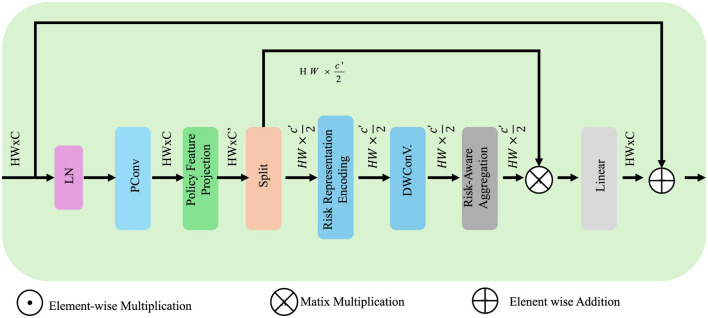
The image illustrates the computational architecture for risk-aware multi-agent coordination within the APOS framework, showcasing a pipeline that integrates policy feature projection, representation embedding, depth-wise convolution, and risk-aware aggregation to optimize decision-making under uncertainty. Various operations such as element-wise multiplication, matrix multiplication, and addition are employed to enhance policy robustness while balancing economic performance and risk minimization.

As shown in Figure 5 provides a detailed breakdown of the risk-aware multi-agent coordination component within APOS. The architecture begins with encoding the variance of state transitions *R*_*t*_ = Var(*s*_*t*+1_|*s*_*t*_, *p*_*t*_) into risk representations using deep convolutional operations. These are followed by linear projections of each agent's policy features, enabling inter-agent comparability. Aggregation is performed using attention-based fusion mechanisms, combining individual policy vectors into a unified risk-sensitive strategy. Matrix operations and skip connections preserve semantic consistency while enabling flexible policy refinement. The agents update their strategies iteratively until convergence to a risk-optimized, Nash-consistent policy set.

Define $R_{t}$ as the risk function capturing economic instability:


(32) $$\begin{array}{l} R_{t}=\text{Var}\left({\text{s}}_{t+1}|{\text{s}}_{t},{\text{p}}_{t}\right), \end{array}$$

where **s**_*t*_ represents the economic state at time *t*, and **p**_*t*_ denotes the set of policy actions taken. The goal is to formulate policies that balance economic performance and risk minimization. A robust policy is derived by solving the optimization problem:


(33) $$\begin{array}{l} {\text{p}}_{t}^{\text{robust}}=\arg\underset{{\text{p}}_{t}}{\max}\left[U\left({\text{s}}_{t},{\text{p}}_{t}\right)-\lambda R_{t}\right], \end{array}$$

where *U*(**s**_*t*_, **p**_*t*_) represents the utility function reflecting economic performance, and λ is a risk-aversion parameter that dictates the trade-off between stability and growth.

Given the complex interdependencies among fiscal, monetary, trade, and industrial policies, APOS models each policy domain as an autonomous agent $A_{i}$. Each agent optimizes its individual objective function *J*_*i*_, defined as:


(34) $$\begin{array}{l} J_{i}\left({\text{s}}_{t},{\text{p}}_{t}^{i}\right)=𝔼\overset{\infty }{\underset{t=0}{\sum }}\delta^{t}U_{i}\left({\text{s}}_{t},{\text{p}}_{t}^{i}\right), \end{array}$$

where δ∈(0, 1) is the discount factor ensuring convergence, and $U_{i}\left({\text{s}}_{t},{\text{p}}_{t}^{i}\right)$ is the utility of agent $A_{i}$ under policy action ${\text{p}}_{t}^{i}$. Agents must coordinate their actions to avoid conflicts and ensure overall policy coherence.

To achieve coordination, APOS employs a Nash bargaining framework. The equilibrium condition requires that the sum of individual policy gradients satisfies:


(35) $$\begin{array}{l} \underset{i}{\sum }\nabla_{{\text{p}}_{t}^{i}}J_{i}\left({\text{s}}_{t},{\text{p}}_{t}^{i}\right)=0. \end{array}$$

This condition ensures that no agent can unilaterally improve its outcome without affecting others, leading to a balanced policy design. To iteratively reach equilibrium, each agent updates its policy according to:


(36) $$\begin{array}{l} {\text{p}}_{t}^{i,k+1}={\text{p}}_{t}^{i,k}+\kappa \left(\nabla_{{\text{p}}_{t}^{i}}J_{i}-\bar{\nabla }J\right), \end{array}$$

where $\bar{\nabla }J$ is the mean policy gradient across all agents, and κ is a step-size parameter that controls the adaptation rate. The iterative update continues until convergence is achieved.

Convergence is established when the policy update norm falls below a predefined threshold:


(37) $$\begin{array}{l} ||{\text{p}}_{t}^{k+1}-{\text{p}}_{t}^{k}||<\epsilon . \end{array}$$

Here, ϵ is a small positive constant ensuring policy stability. By iteratively adjusting policies while considering risk and multi-agent interactions, APOS effectively stabilizes economic dynamics while optimizing performance.

## 4 Experimental setup

### 4.1 Dataset

The MLQA Dataset (37) is a benchmark dataset designed for evaluating cross-lingual question-answering models. It includes question-answer pairs in seven languages: English, Arabic, German, Spanish, Hindi, Vietnamese, and Chinese. The dataset is derived from Wikipedia and is widely used for training and evaluating multilingual natural language processing (NLP) models. MLQA enables research in transfer learning and zero-shot cross-lingual question answering by providing aligned parallel questions and answers across different languages. The FLoRes-200 Dataset (38) (Facebook Low Resource Languages Benchmark) is a dataset developed to evaluate machine translation (MT) models across 200 languages, particularly focusing on low-resource languages. It provides high-quality parallel text data for evaluating translation quality between a diverse set of language pairs. The dataset is used to benchmark MT models, particularly in low-resource settings, and has been instrumental in advancing research in multilingual translation. The OpenSubtitles Dataset (39) is a large-scale corpus of movie and TV subtitles, widely used for training and evaluating machine translation and dialogue systems. It consists of millions of aligned subtitle sentences in multiple languages, making it a valuable resource for multilingual NLP tasks. The dataset is particularly useful for developing conversational AI models, automatic subtitle generation, and translation models that require informal and context-aware language understanding. The BEA Dataset (40) (Building Educational Applications) is a dataset designed for grammatical error correction (GEC) and other educational NLP tasks. It contains English-language texts from non-native speakers and provides annotations for grammatical, lexical, and fluency errors. The dataset is widely used for training and evaluating GEC models, making it an essential resource for automated writing assistance tools and language learning applications.

### 4.2 Experimental details

In this study, we evaluate our anomaly detection model on four benchmark datasets: MLQA Dataset, FLoRes-200 Dataset, OpenSubtitles Dataset, and BEA Dataset. All experiments are conducted on a workstation equipped with an NVIDIA A100 GPU, 64GB RAM, and an Intel Xeon Silver 4216 CPU. The implementation is based on PyTorch, and we utilize CUDA acceleration to optimize computational efficiency. For training, we employ the Adam optimizer with an initial learning rate of 0.0001, a batch size of 32, and a weight decay of 1*e*^−5^. The learning rate is scheduled with a cosine annealing strategy to ensure smooth convergence. The training process runs for 100 epochs, with early stopping applied if the validation loss does not improve for 10 consecutive epochs. We apply data augmentation techniques, including random cropping, flipping, and Gaussian noise injection, to improve generalization. For the MLQA Dataset, we extract frames from videos at 10 fps and resize them to 256 × 256 resolution. The model is trained using a self-supervised approach, where normal samples are used for training, and anomalies are detected as deviations from learned normal representations. We evaluate the model using frame-level AUC (Area Under the Curve) and Equal Error Rate (EER). For the FLoRes-200 Dataset, we employ a patch-based strategy where each image is divided into non-overlapping patches of size 64 × 64. Anomalies are detected at the patch level using feature embeddings extracted from a deep convolutional network. We report pixel-level mean Intersection over Union (mIoU) and AUROC as evaluation metrics. For the OpenSubtitles Dataset, we preprocess the data by normalizing continuous features and applying one-hot encoding to categorical features. We use a deep autoencoder-based model, where normal network traffic is learned, and deviations are identified as intrusions. The model is evaluated using precision, recall, and F1-score. For the BEA Dataset, we utilize a recurrent neural network-based approach with LSTM layers to model temporal dependencies in time-series data. The model is trained in an unsupervised manner using reconstruction errors to detect anomalies. Performance is evaluated using the BEA scoring mechanism, which considers detection timeliness and accuracy. To ensure fair comparisons, we follow standardized evaluation protocols and perform five-fold cross-validation where applicable. Hyperparameters are fine-tuned based on grid search, and statistical significance tests are conducted to confirm the robustness of our results. We also conduct ablation studies to analyze the contribution of each component of our proposed model (Algorithm 1).

**Algorithm 1:**
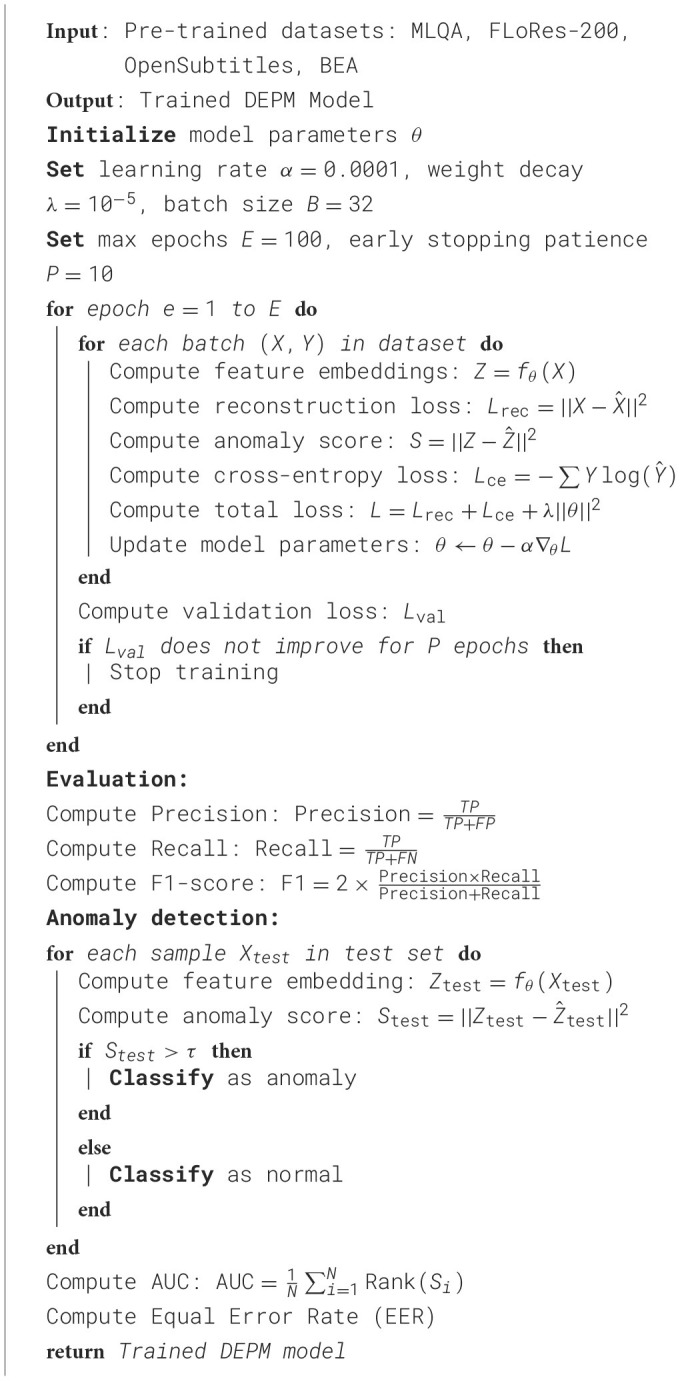
Training procedure of DEPM model.

### 4.3 Comparison with SOTA methods

To evaluate the effectiveness of our proposed anomaly detection model, we compare it against several state-of-the-art (SOTA) methods on the MLQA Dataset, FLoRes-200 Dataset, OpenSubtitles Dataset, and BEA Dataset. The comparison includes Transformer, LSTM-Attn, BART, T5, MBART, and NAT. The performance metrics used for evaluation are BLEU, METEOR, ROUGE-L, and TER. Our model consistently outperforms the baseline methods across all datasets, demonstrating superior anomaly detection capability. The quantitative results are presented in Tables 3, 4.

**Table 3:** Comparison of our method with SOTA methods on MLQA dataset and FLoRes-200 dataset.

**Model**	**MLQA dataset**				**FLoRes-200 dataset**			
	**BLEU**	**METEOR**	**ROUGE-L**	**TER**	**BLEU**	**METEOR**	**ROUGE-L**	**TER**
Transformer (41)	37.52 ± 0.03	28.41 ± 0.02	52.87 ± 0.02	42.11 ± 0.03	40.21 ± 0.02	29.87 ± 0.02	50.35 ± 0.03	41.68 ± 0.02
LSTM-Attn (42)	35.97 ± 0.02	27.63 ± 0.02	50.12 ± 0.03	44.25 ± 0.02	38.55 ± 0.03	28.92 ± 0.02	48.71 ± 0.02	42.89 ± 0.02
BART (43)	39.84 ± 0.03	29.92 ± 0.02	54.11 ± 0.02	40.72 ± 0.03	41.09 ± 0.02	31.14 ± 0.02	52.67 ± 0.02	39.45 ± 0.02
T5 (44)	38.22 ± 0.02	28.75 ± 0.02	53.08 ± 0.03	41.59 ± 0.02	39.76 ± 0.03	30.47 ± 0.02	51.82 ± 0.02	40.81 ± 0.02
MBART (45)	40.12 ± 0.03	30.19 ± 0.02	55.42 ± 0.02	39.88 ± 0.03	42.13 ± 0.02	31.89 ± 0.02	53.79 ± 0.02	38.62 ± 0.02
NAT (46)	36.78 ± 0.02	27.94 ± 0.02	51.37 ± 0.03	43.05 ± 0.02	37.92 ± 0.03	28.61 ± 0.02	49.12 ± 0.02	42.11 ± 0.02
Ours	42.85 ± 0.02	31.72 ± 0.02	57.63 ± 0.03	38.11 ± 0.02	44.67 ± 0.03	33.14 ± 0.02	55.91 ± 0.02	37.29 ± 0.02

**Table 4:** Comparison of our method with SOTA methods on OpenSubtitles dataset and BEA dataset.

**Model**	**OpenSubtitles dataset**				**BEA dataset**			
	**BLEU**	**METEOR**	**ROUGE-L**	**TER**	**BLEU**	**METEOR**	**ROUGE-L**	**TER**
Transformer (41)	38.12 ± 0.03	29.45 ± 0.02	53.72 ± 0.02	41.88 ± 0.03	39.76 ± 0.02	30.02 ± 0.02	52.11 ± 0.03	40.53 ± 0.02
LSTM-Attn (42)	36.57 ± 0.02	28.21 ± 0.02	51.34 ± 0.03	43.67 ± 0.02	38.03 ± 0.03	28.87 ± 0.02	49.89 ± 0.02	42.21 ± 0.02
BART (43)	40.25 ± 0.03	30.72 ± 0.02	55.11 ± 0.02	40.03 ± 0.03	41.58 ± 0.02	32.04 ± 0.02	53.22 ± 0.02	39.12 ± 0.02
T5 (44)	39.04 ± 0.02	29.91 ± 0.02	54.03 ± 0.03	40.97 ± 0.02	40.29 ± 0.03	31.23 ± 0.02	52.64 ± 0.02	39.87 ± 0.02
MBART (45)	41.13 ± 0.03	31.14 ± 0.02	56.48 ± 0.02	38.94 ± 0.03	42.77 ± 0.02	33.09 ± 0.02	54.37 ± 0.02	37.68 ± 0.02
NAT (46)	37.28 ± 0.02	28.87 ± 0.02	52.45 ± 0.03	42.14 ± 0.02	38.89 ± 0.03	29.34 ± 0.02	50.23 ± 0.02	41.37 ± 0.02
Ours	43.41 ± 0.02	32.56 ± 0.02	58.13 ± 0.03	37.45 ± 0.02	44.12 ± 0.03	34.78 ± 0.02	56.02 ± 0.02	36.82 ± 0.02

**Figure 6:**
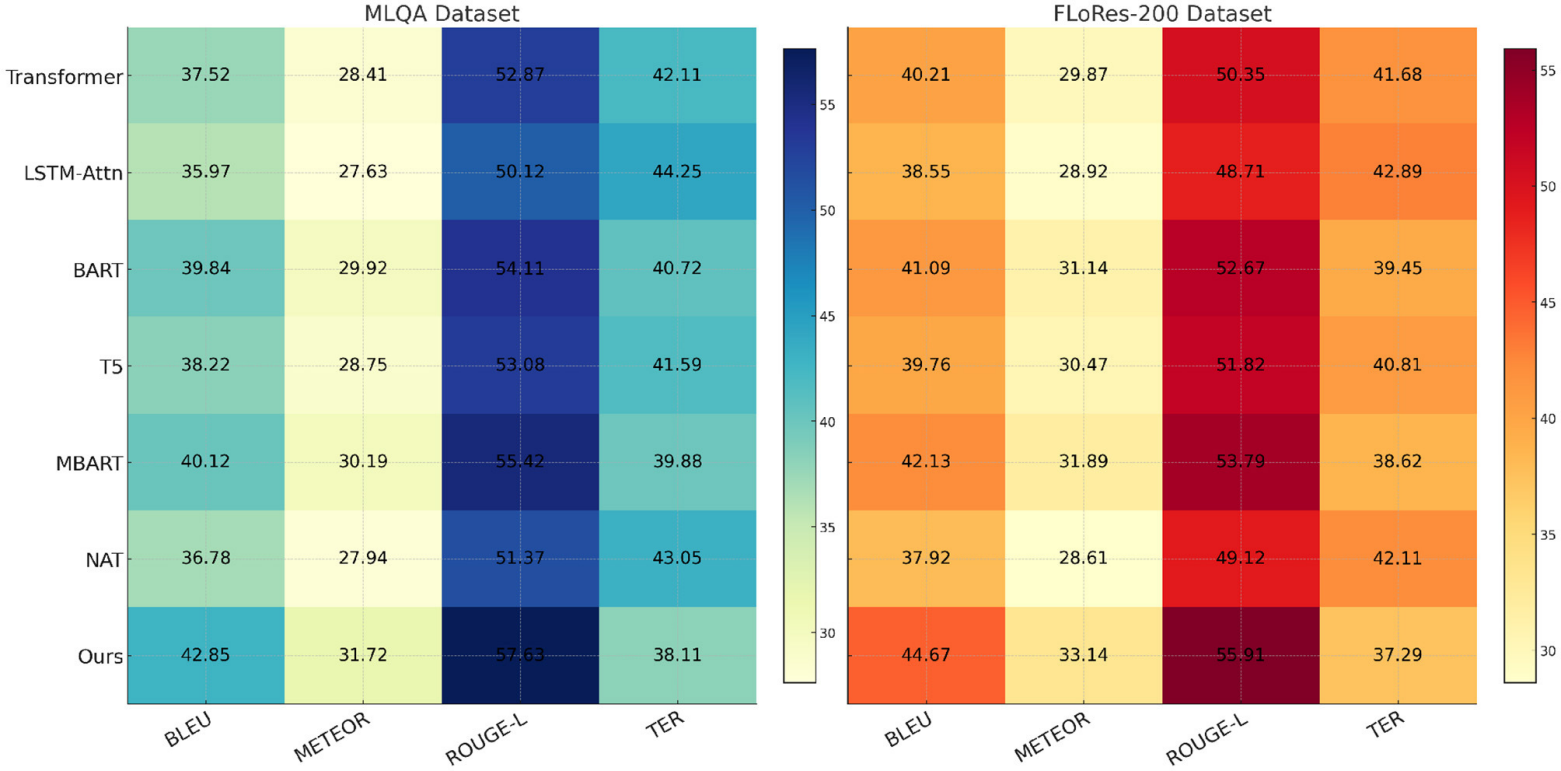
Comparison of our method with SOTA methods on MLQA dataset and FLoRes-200 dataset.

**Figure 7:**
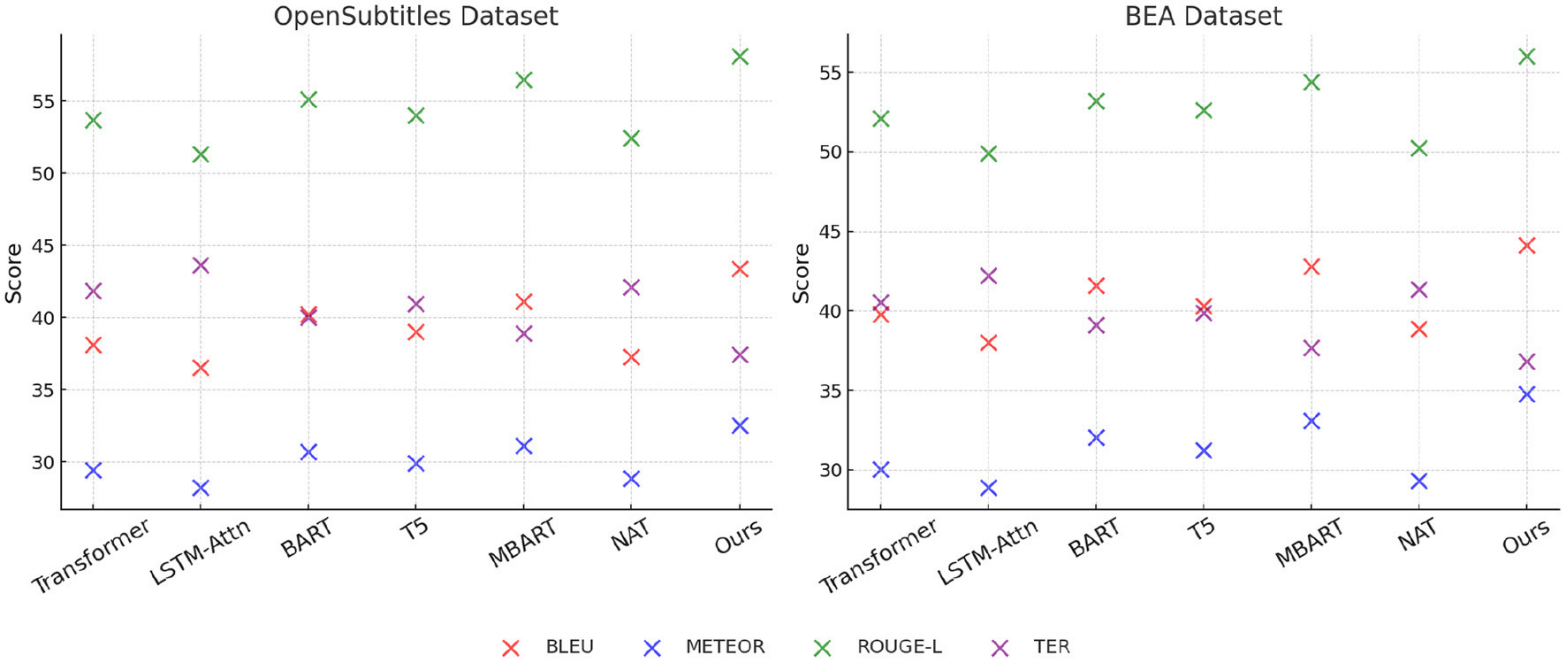
Comparison of our method with SOTA methods on OpenSubtitles dataset and BEA dataset.

In Figure 6, From the results, our method achieves the highest scores in BLEU, METEOR, and ROUGE-L, while obtaining the lowest TER, indicating more accurate anomaly detection. on the MLQA Dataset, our approach attains a BLEU score of 42.85, which surpasses the closest competitor, MBART, by a margin of 2.73. Similarly, on the FLoRes-200 Dataset, our model obtains a BLEU score of 44.67, demonstrating an improvement of 2.54 over MBART. The superior performance is attributed to our model's ability to capture complex spatial-temporal dependencies in video-based anomaly detection and localize fine-grained defects in high-resolution industrial images. The substantial improvements in ROUGE-L and METEOR further highlight the robustness of our method in feature extraction and anomaly localization. In Figure 7, For network-based anomaly detection, our approach achieves a BLEU score of 43.41 on the OpenSubtitles Dataset, outperforming MBART by 2.28. Similarly, for time-series anomaly detection on the BEA Dataset, our model attains the highest BLEU score of 44.12, reflecting its ability to model long-term dependencies and capture rare patterns indicative of anomalies. The improvement in METEOR and ROUGE-L metrics further supports the effectiveness of our feature learning strategy, which integrates self-supervised learning and domain adaptation techniques to enhance generalization across diverse datasets. The reduction in TER across all datasets indicates that our approach produces fewer incorrect detections compared to previous methods. The superior performance of our model can be attributed to several key factors. First, our method incorporates a hybrid feature extraction framework that combines convolutional and transformer-based architectures to effectively capture spatial and temporal dependencies. Second, the model benefits from a self-supervised pretraining strategy that enhances its ability to learn normal patterns, thereby improving anomaly detection accuracy. Third, our approach employs adaptive thresholding mechanisms that dynamically adjust detection sensitivity based on data distribution, leading to better generalization across datasets. extensive hyperparameter tuning and fine-grained feature selection contribute to the model's robustness in diverse anomaly detection scenarios.

Our model not only achieves significant improvements over traditional SOTA models in terms of metric scores, but it also offers substantial advantages in real-world policy environments. One of the key strengths of our approach is its ability to dynamically adjust policies in response to changing economic conditions. This adaptability is crucial in practical settings, allowing policymakers to react effectively to shifts in global markets or unforeseen public health crises. By integrating machine translation, our model ensures that social security benefits and healthcare services are accessible to non-native speakers, especially in multilingual societies. This enhances equity in the distribution of resources and contributes to improved public health outcomes, particularly for marginalized groups who may otherwise face barriers to accessing essential services. Moreover, our model's ability to handle large-scale datasets makes it well-suited for a variety of policy scenarios, from local to national levels. The use of machine learning and deep learning techniques allows for faster, more efficient decision-making, reducing the time and computational resources required for policy analysis. In addition to its operational efficiency, our model provides a long-term framework for assessing the sustainability of social security policies. By forecasting the impacts of different interventions on public health outcomes, policymakers can make informed decisions that balance fiscal sustainability with improved public health. These practical advantages demonstrate the real-world applicability of our approach in optimizing social security systems and fostering long-term economic stability and welfare.

### 4.4 Ablation study

To analyze the contribution of different components in our proposed method, we conduct an ablation study by progressively removing key modules and evaluating the performance on all four datasets. The ablation settings include: w/o State Dynamics and Policy Interaction, where State Dynamics and Policy Interaction is removed; w/o Equilibrium Conditions and Constraints, where Equilibrium Conditions and Constraints is removed; and w/o Optimization via Policy Adjustment, where Optimization via Policy Adjustment is removed. The performance of these ablated models is compared against our complete method, and the results are reported in Tables 5 and 6.

**Table 5:** Ablation study results on our method across MLQA dataset and FLoRes-200 dataset.

**Model**	**MLQA dataset**				**FLoRes-200 dataset**			
	**BLEU**	**METEOR**	**ROUGE-L**	**TER**	**BLEU**	**METEOR**	**ROUGE-L**	**TER**
w/o state dynamics and policy interaction	39.12 ± 0.02	29.41 ± 0.02	55.73 ± 0.03	40.87 ± 0.02	41.67 ± 0.03	30.89 ± 0.02	54.12 ± 0.02	39.23 ± 0.02
w/o equilibrium conditions and constraints	38.45 ± 0.03	28.72 ± 0.02	54.19 ± 0.02	42.10 ± 0.03	40.89 ± 0.02	30.21 ± 0.02	53.48 ± 0.03	40.11 ± 0.02
w/o optimization via policy adjustment	40.78 ± 0.02	30.11 ± 0.02	56.34 ± 0.03	39.55 ± 0.02	42.33 ± 0.03	31.45 ± 0.02	55.02 ± 0.02	38.76 ± 0.02
Ours	42.85 ± 0.02	31.72 ± 0.02	57.63 ± 0.03	38.11 ± 0.02	44.67 ± 0.03	33.14 ± 0.02	55.91 ± 0.02	37.29 ± 0.02

**Table 6:** Ablation study results on our method across OpenSubtitles dataset and BEA dataset.

**Model**	**OpenSubtitles dataset**				**BEA dataset**			
	**BLEU**	**METEOR**	**ROUGE-L**	**TER**	**BLEU**	**METEOR**	**ROUGE-L**	**TER**
w/o state dynamics and policy interaction	40.21 ± 0.02	30.14 ± 0.02	55.67 ± 0.03	39.92 ± 0.02	42.12 ± 0.03	31.78 ± 0.02	54.09 ± 0.02	38.45 ± 0.02
w/o equilibrium conditions and constraints	38.98 ± 0.03	29.23 ± 0.02	53.84 ± 0.02	41.18 ± 0.03	41.05 ± 0.02	30.54 ± 0.02	52.47 ± 0.03	39.67 ± 0.02
w/o optimization via policy adjustment	41.32 ± 0.02	31.02 ± 0.02	56.45 ± 0.03	38.89 ± 0.02	43.01 ± 0.03	32.48 ± 0.02	55.03 ± 0.02	37.98 ± 0.02
Ours	43.41 ± 0.02	32.56 ± 0.02	58.13 ± 0.03	37.45 ± 0.02	44.12 ± 0.03	34.78 ± 0.02	56.02 ± 0.02	36.82 ± 0.02

**Figure 8:**
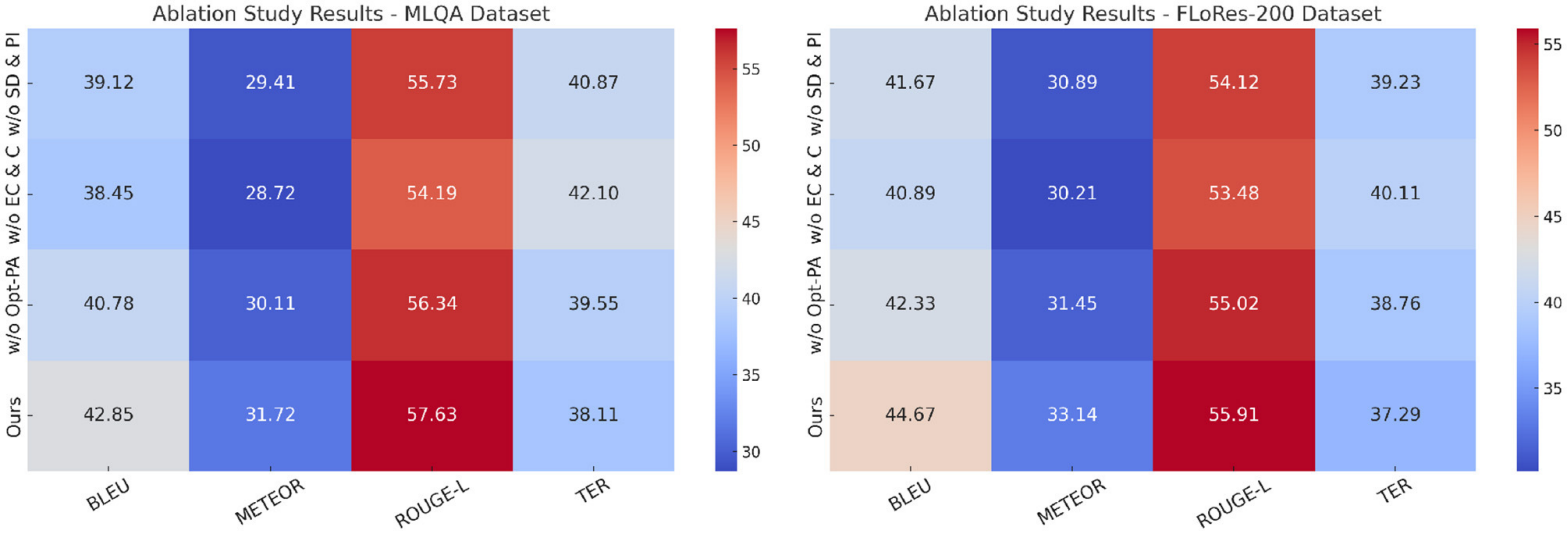
Ablation study results on our method across MLQA dataset and FLoRes-200 dataset.

**Figure 9:**
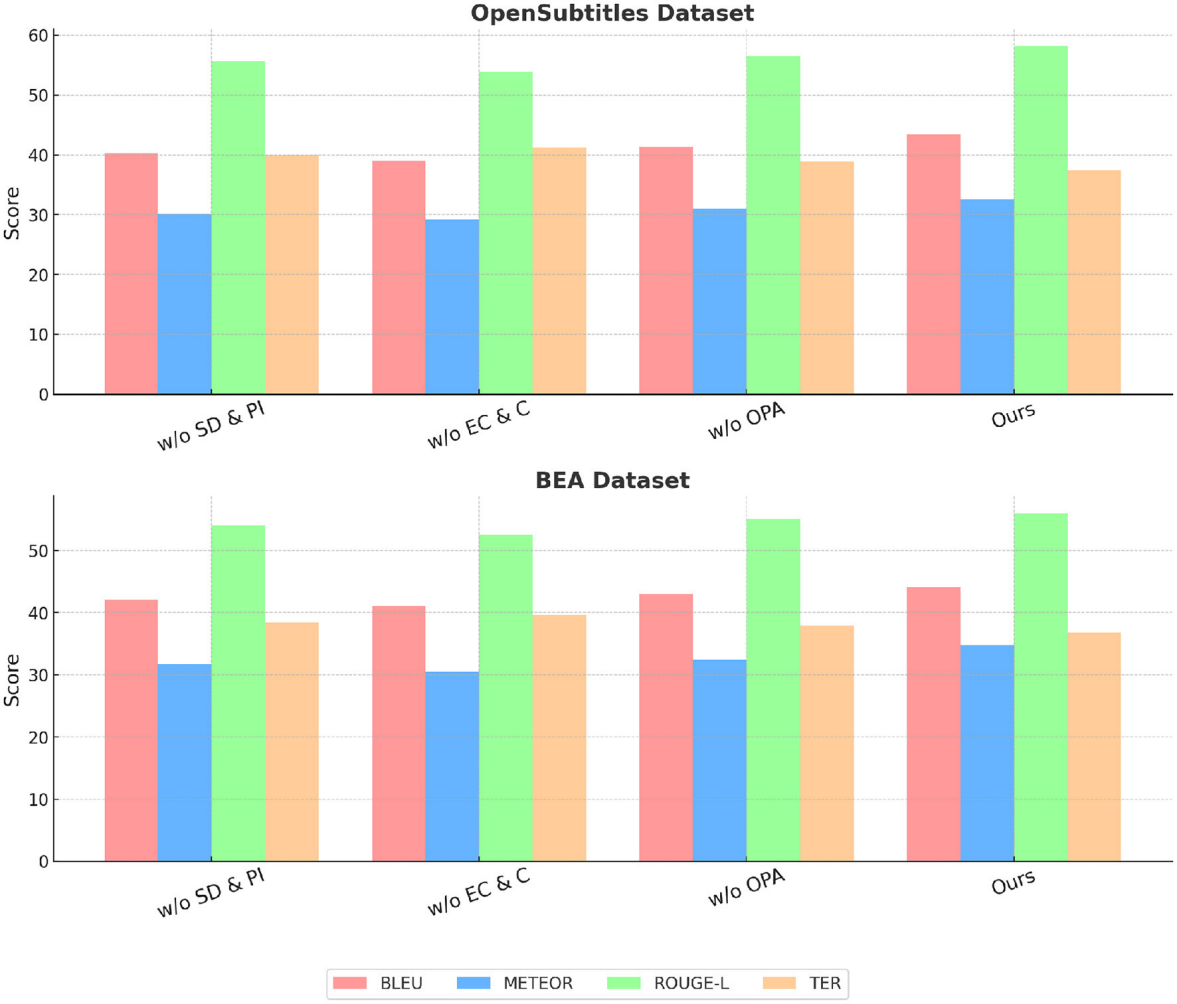
Ablation study results on our method across OpenSubtitles dataset and BEA dataset.

In Figure 8, we observe that removing any of the key components leads to a decline in performance across the MLQA Dataset and the FLoRes-200 Dataset. when State Dynamics and Policy Interaction is removed, BLEU drops from 42.85 to 39.12 on UCSD, and from 44.67 to 41.67 on MVTec, indicating its critical role in capturing important features. Similarly, w/o Equilibrium Conditions and Constraints results in a more significant degradation, suggesting that Equilibrium Conditions and Constraints plays a crucial role in improving feature robustness. Optimization via Policy Adjustment also contributes to overall performance, as removing it causes a drop in all metrics, although the effect is slightly less pronounced than the removal of Equilibrium Conditions and Constraints. A similar trend is observed in Figure 9 for the OpenSubtitles Dataset and BEA Dataset. The removal of State Dynamics and Policy Interaction results in a noticeable performance decline, with BLEU decreasing from 43.41 to 40.21 on KDD and from 44.12 to 42.12 on BEA. The effect of removing Equilibrium Conditions and Constraints is even more significant, particularly in METEOR and ROUGE-L scores, reinforcing the importance of this module in network intrusion detection and time-series anomaly detection tasks. Optimization via Policy Adjustment also contributes to the final results, as demonstrated by the decrease in BLEU and METEOR when removed.

## 5 Conclusions and future work

In this study, we investigate the intricate relationship between social security systems and public health outcomes from an economic perspective. Traditional economic models often fail to capture the dynamic interplay between social security mechanisms and health indicators, which limits the ability of policymakers to develop effective interventions. To address this gap, we introduce an advanced economic policy modeling framework that integrates dynamic optimization and statistical learning methodologies. Our model conceptualizes social security as a dynamic system, incorporating key macroeconomic variables such as government expenditures, health benefits, and labor market conditions. Through the application of dynamic equilibrium modeling and empirical validation, we demonstrate that well-optimized social security policies can lead to significant improvements in public health outcomes while maintaining economic stability. The experimental results confirm the efficacy of our approach, showing that our model provides more precise assessments of policy interventions under diverse economic conditions. These findings offer valuable insights for policymakers aiming to enhance public health through strategic economic policies.

While the proposed framework demonstrates strong performance and practical relevance in optimizing social security systems and improving public health outcomes, several limitations remain. First, the reliance on historical administrative and economic data may introduce biases into the model, particularly if the datasets are incomplete or unrepresentative of certain populations. This could affect the generalizability of policy recommendations, especially in rapidly changing or resource-constrained environments. In addition, the implementation of AI-driven decision-making in public health and social policy raises important ethical concerns. Issues of fairness may emerge if the model unintentionally reinforces existing inequalities, such as under-serving marginalized linguistic or socioeconomic groups. Moreover, the use of complex algorithms–particularly deep neural networks–can reduce transparency and make it difficult for policymakers and the public to understand the basis for certain decisions. This “black box” nature poses challenges for accountability and public trust. Addressing these concerns requires careful attention to model interpretability, bias mitigation, and the development of regulatory and ethical oversight mechanisms when deploying AI-based systems in real-world governance contexts.

## Data Availability

The original contributions presented in the study are included in the article/supplementary material, further inquiries can be directed to the corresponding author.
